# Recommendations for physician appointments of LGBTQIA+ adolescents with chronic conditions

**DOI:** 10.1016/j.clinsp.2024.100507

**Published:** 2024-10-19

**Authors:** Pedro Henrique P. Sampaio, Darusa C. Souza, Pedro Henrique M. Mendes, Ligia B. Queiroz, Benito Lourenço, Marcia T. Couto, Clovis A. Silva

**Affiliations:** a Adolescent Unit, Instituto da Criança e Adolescente, Hospital das Clínicas da Faculdade de Medicina da Universidade de São Paulo (HCFMUSP), São Paulo, SP, Brazil; b Department of Preventive Medicine, Faculdade de Medicina, Universidade de São Paulo (FMUSP), São Paulo, SP, Brazil

Enhanced management of infectious diseases, improvement in health-related quality of life, nutrition, and hygiene, along with increased life expectancy, are driving an epidemiological transition among adolescents. This transition is marked by the increase of pediatric chronic diseases as significant health issues among youths.^1^^,^^2^ Numerous physical, emotional, and behavioral changes occur during adolescence, and an additional long-term illness can delay growth, puberty, sexuality and the maturation of other biological systems.

A notable increase in knowledge regarding many chronic conditions across various pediatric specialties has been reported in recent years. These advancements in children and adolescents’ chronic conditions have been particularly observed in Adolescent Medicine, Allergy and Immunology, Cardiology, Endocrinology, Gastroenterology, Genetics, Hematology, Transplantation Care, Hepatology, Infectious Diseases, Nephrology, Neurology, Nutrition, Oncology, Orthopedics, Palliative and Pain Care, Pediatric Surgery, Pulmonology, Premature Infant Care, Psychiatry, Rheumatology, and other fields.^1^^,^^2^ However, when a health-threatening condition, such as a chronic illness, overlaps with the challenges faced by an LGBTQIA+ (Lesbian, Gay, Bisexual, Transgender, Queer, Intersex, Asexual, and others) adolescent at this stage of life, it significantly amplifies the risk and vulnerability to psychological distress. This overlap also raises important questions about both mental and physical health, as well as health-related quality of life. Therefore, it is important to recognize that LGBTQIA+ adolescents dealing with chronic illnesses may experience higher levels of psychological distress due to stigma and discrimination. Consequently, they may face additional barriers in the context of healthcare and treatment.

Studies of LGBTQIA+ adolescents and youths have shown that they may experience a higher prevalence of HIV and other sexually transmitted infections, bullying, physical abuse, anxiety, and depression.^3^ A systematic review and meta-analysis study revealed that among adolescent sexual minority males who had engaged in sexual activity within the past 6-months or were sexually active, 44 % reported engaging in anal intercourse without condom use, 50 % did not use a condom during their last sexual encounter, and 32 % reported using alcohol or drugs at their last sexual experience.^4^ When addressing the risk of HIV infection among adolescents and young people, especially LGBTQIA+ individuals, combined prevention strategies, particularly the availability of Pre-Exposure Prophylaxis (PrEP), have proven to be effective preventive measures. However, PrEP was more commonly accessed by adolescents and young men who have sex with men.^5^ Another study focusing on adolescent and young gay and bisexual men identified substantial barriers to accessing and adhering to PrEP, particularly influenced by social factors such as race and socioeconomic status. Using an intersectional approach, the study highlighted the significance of considering the subjective and relational experiences of young people in the development of HIV prevention strategies. It emphasized the necessity for a more inclusive approach informed by PrEP users themselves to enhance the effectiveness of HIV prevention programs and overall healthcare delivery.^6^

Sexual and gender minorities also reported more frequent polysubstance use than non-minorities.^7^ Furthermore, transgender and gender-diverse individuals should have prompt access to healthcare services. Treatments for gender transition such as gonadotropin-releasing hormone agonists and gender-affirming hormones may impact growth, as well as bone, cardiovascular, and reproductive health.^8^ These points are also crucial to discuss and individualize the approach to address these issues for each LGBTQIA+ adolescent with a chronic condition.

Another significant finding is that only a small number of physicians providing LGBTQIA+ care to adolescents felt sufficiently equipped to address the needs of their sexual-minority patients due to a lack of education and resources.^9^ Moreover, some LGBTQIA+ adolescents with chronic conditions may refrain from attending multiprofessional and multidisciplinary appointments due to concerns about facing discrimination and anticipated stigma. These findings may significantly impact adolescent adherence to medications and rehabilitation programs, potentially resulting in disease flares, damage, and complications of pediatric chronic conditions.

Pediatricians should have offices that are teen-friendly and welcoming to all adolescents, regardless of sexual orientation and behavior.^10^ Adolescent Health and Medicine Society supports that all healthcare providers who care for adolescents should be trained to provide competent and nonjudgmental care for LGBTQIA+ youth.^11^ Indeed, the Adolescent Unit of our university and tertiary hospital has been providing care for LGBTQIA+ adolescents and their families with both healthy and chronic conditions over the past several years, focusing on a person-centered care approach. However, further qualitative and quantitative studies involving LGBTQIA+ teens with chronic conditions will be essential to fully understand and address their specific needs.

It is essential to standardize the systematization and dynamics of first and further medical appointments for LGBTQIA+ adolescents with chronic illnesses, and the authors suggest three or two steps. As outlined in Table 1, the first medical appointment for adolescents and family members with chronic illnesses should follow a structured approach consisting of three or two steps. Within our group, the authors recommend that the initial medical appointment be structured into three distinct steps. During the first step, healthcare providers should explain the dynamics of care, emphasizing aspects of privacy, confidentiality, accessibility, collaborative approach, and non-discrimination language and policies, and a care oriented to the adolescent´s ability to make informed decisions as they grow older. The second stage of the first appointment involves a session only with the adolescent's family members, aiming to gather the family and youth's medical history. This stage allows an opportunity for parents and caregivers to express their fears and concerns to the general or pediatric specialty physician, while also creating a supportive environment for them to discuss their own experiences. Finally, the third step is dedicated solely to the adolescents, providing them with a safe space to express themselves freely and discuss any further worries or questions they may have. This includes topics such as sexual and emotional development, as well as issues related to their chronic condition. Further appointments for teens with an adolescent medicine specialist or another pediatric specialty physician will be divided into two steps (Table 1). The physical examination and laboratory assessment of LGBTQIA+ adolescents with chronic illnesses are performed in the same manner as for any adolescent. Importantly, this also includes attention to signs and symptoms specific to each adolescent's chronic condition, ensuring comprehensive and tailored healthcare delivery.

**Table 1:** Recommendations for physician appointment of LGBTQIA+ (Lesbian, Gay, Bisexual, Transgender, Queer, Intersex, Asexual, and Others) adolescent with chronic conditions.

Recommendations	Appointment of LGBTQIA+ adolescent with chronic condition
**First appointment in three or two steps**	**Three moments**
	**First step:** Family members and adolescent
	**Second step:** Only family members
	**Third step:** Only adolescent
	**OR**
	**Two moments**
	**First step:** Family members and adolescent
	**Second step:** Only adolescent
**Further appointments in two steps**	**First step:** Family members and adolescent
	**Second step:** Only adolescent
**Medical confidentiality, privacy, accessibility, collaborative approach, and non-discrimination policies**	These points are explained in detail during the first step of the initial appointment and are reiterated whenever questioned to ensure clarity. A nonjudgmental healthcare environment and effective communication skills are essential.
**Establishing a connection between physician and adolescent**	Establishing a safe and non-judgmental space is crucial. The discussion should occur in a confidential environment where teens feel secure and respected. Empathy, respect for autonomy and open communication without judgment are encouraged in all appointments.
**Inquiring about family data and dynamics**	Where is your place of residence?
	Who shares the household space with you?
	Is the environment characterized by peacefulness or conflicts?
	How do interpersonal relationships function in your home?
**Inquiring about school and social impact**	Do you feel safe at school and in your neighborhood?
	Have you experienced bullying, physical aggression, or verbal threats?
	Do you have a group of friends?
	Do you ever feel lonely or without friends?
	Do you experience any difficulties with learning or academic performance?
**Inquiring about dietary habits and body image issues**	Have you ever tried any type of dietary regimen?
	Have you experimented with restrictive diets or prolonged fasting?
	Do you experience episodes of binge eating?
	Are you satisfied with your physical appearance/body?
	Do you feel comfortable with your current weight and height?
**Inquiring about drug and psychoactive substance use**	To assess the risk and vulnerability related to legal substances such as alcohol, tobacco, e-cigarettes, hookah, as well as illegal substances:
	Do you consume alcoholic beverages? If yes, how often?
	When was the last time you drank excessively?
	Where do you usually consume alcohol? (e.g., at home, in bars, at parties)
	Do your friends often engage in heavy drinking?
	Does anyone in your home consume alcoholic beverages?
**Inquiring about sexual behaviors, gender identity, sexual orientation, reproductive health, incidence of unplanned pregnancies, and Sexually Transmitted Infections (STIs)**	Are you currently involved in a romantic relationship?
	Do you feel in love with anyone now?
	Are you engaging in physical intimacies with anyone?
	Have you had sexual experiences with individuals of the opposite sex, the same sex, or do you have no specific preference?
	Have you ever had any reproductive health concerns or issues?
	Have you experienced any unplanned pregnancies?
	Have you ever been tested for HIV and other Sexually Transmitted Infections (STIs)?
	Have you ever been diagnosed with an STI?
	Are you currently using any methods of contraception?
	Would you typically use daily HIV Pre-Exposure Prophylaxis (PrEP) or post-exposure prophylaxis to prevent HIV when engaging in sexual activities without a condom?
**Inquiring about mental health**	How do you typically cope with feelings of sadness or distress?
	Do you prefer to be alone in silence when you're feeling sad, or do you seek comfort from others?
	Do you find yourself crying often when you're feeling down?
	Have you ever experienced thoughts of wanting to disappear or hurt yourself?
	Have you ever attempted to hurt yourself before?
	In the past two weeks, have you felt little interest or pleasure in doing activities that you usually enjoy?
	Have you felt consistently down, depressed, or hopeless recently? If so, how often?
**Inquiring about drugs, vaccination, and other treatment adherence**	Where and how do you usually take your prescribed drugs and treatments?
	Do you have assistance from anyone in managing your drugs and treatments? If so, who helps you?
	Is there any supervision or oversight involved in your medication regimen?
	How does the responsibility for managing your medications and treatments feel for you?
	How do you perceive yourself in the role of being ill versus not ill?
	Has your vaccination card been updated?
**Inquiring about psychology or psychiatric support**	Are you currently receiving psychological or psychiatric support?
	If yes, how long have you been receiving therapy?
	Was your therapy individual or group-based?
	Do you know which approach your therapy aligns with? (e.g., cognitive-behavioral therapy, psychoanalysis)
	Do you feel you have a good rapport with your psychologist or psychiatrist?
**Inquiring about spirituality and religion**	Do you practice any religion or attend religious services? If yes, how often?
	What are your beliefs regarding spirituality or religion?
	Do you engage in practices such as prayer or meditation?
	Have you ever experienced existential conflicts or concerns about death?
**Violence**	Physical and sexual violence are issues common among adolescents and pose a significant risk to their safety and well-being. When it comes to gender and sexual orientation-related violence, interventions for violence prevention need to be increased to actively address factors linked to violence during adolescence. Approaching this problem with sensitivity is important, as a suffering teenager may find it difficult to initiate a conversation about such a sensitive issue.

Clinicians should remember that distinct racial/ethnic, religious, and demographic groups may have stressors for LGBTQIA+ adolescents with chronic illnesses. The experience of “coming out' for LGBTQIA+ teens may also be discussed during the appointment. It may be a constructive experience for some adolescents, fostering acceptance and a sense of liberation. However, for others, it can be harmful, resulting in stigma, humiliation, peer rejection, discrimination, and ostracism, which can consequently lead to significant emotional issues.^11^ Suicide and hospital admissions for suicide-related thoughts and behaviors are relevant issues for LGBTQIA+ adolescents, particularly for those with chronic conditions. Schools may also offer a unique opportunity to support suicide prevention with the promotion of positive social relationships and fostering a safe and inclusive community environment.^12^

In conclusion, it is vital to prioritize awareness and comprehension of the distinctive and specific requirements of LGBTQIA+ teens with chronic conditions. Nonjudgmental multiprofessional and multidisciplinary clinics, characterized by effective communication skills and devoid of biases, along with emotional care, support groups, and community resources, are mandatory for delivering high-quality care to this population.

## Declaration of competing interest

The authors declare no conflicts of interest.
